# Adaptability and Germination Characteristics of Volunteer Wheat in China’s Main Wheat-Producing Areas

**DOI:** 10.3390/biology12081090

**Published:** 2023-08-04

**Authors:** Wangcang Su, Muhan Yang, Ronghui Ma, Qingqing Li, Hongle Xu, Fei Xue, Lanlan Sun, Chuantao Lu, Renhai Wu

**Affiliations:** Henan Key Laboratory of Crop Pest Control, Institute of Plant Protection, Henan Academy of Agricultural Sciences, Zhengzhou 450002, China; suwangcang@126.com (W.S.); yang521muhan@163.com (M.Y.); mwh222@126.com (R.M.); qingqingli666@163.com (Q.L.); xuhongle86@126.com (H.X.); justxhl@163.com (F.X.); sunjgs@126.com (L.S.)

**Keywords:** seeding emergence, temperature, salt stress, osmotic potential, seeding depth, seed longevity

## Abstract

**Simple Summary:**

Volunteer wheat is commonly found in the main wheat-producing areas of China and impacts cultivated wheat production. This study’s aim was evaluating the effects of environmental conditions on the adaptability and germination of volunteer wheat grains. Our results showed that volunteer wheat was more adaptable to low temperatures of 5 °C and was poorly adaptable to high temperatures of above 30 °C. Volunteer wheat was more adaptable to higher salinity and lower osmotic potential than cultivated wheat. The secondary germination ability of volunteer wheat was also higher than that of cultivated wheat after water immersion. The deep tillage of cultivated land could effectively prevent the spread of volunteer wheat. Our study provides a basis for future studies concerning the control of volunteer wheat.

**Abstract:**

Volunteer wheat commonly occurs and spreads rapidly in the main wheat-producing areas of China, seriously impacting cultivated wheat production. Limited information is currently available regarding the adaptability and germination traits of volunteer wheat. Therefore, this study’s aim was to evaluate the effects of environmental conditions on the germination and emergence of volunteer wheat seeds through laboratory experiments. The results showed that the germination percentages and viability of volunteer wheat were significantly higher than those of cultivated wheat at a low temperature of 5 °C, and they were lower than those of cultivated wheat at high temperatures of above 30 °C. Compared to cultivated wheat, volunteer wheat was able to tolerate higher salinity and lower osmotic potential, especially long-dormancy volunteer wheat. The secondary germination ability of volunteer wheat was higher than that of cultivated wheat after water immersion. Furthermore, volunteer wheat could not emerge normally when the seeding depth was greater than 8 cm, and the emergence ability of the volunteer wheat was weaker than that of the cultivated wheats when the seeding depth was 4–8 cm, which indicates that the deep tillage of cultivated land could effectively prevent the spread of volunteer wheat. This study revealed differences in the germination characteristics of volunteer wheat and cultivated wheat under the influence of different environmental factors, which provides a basis for future studies concerning the control of volunteer wheat.

## 1. Introduction

**Figure 1:**
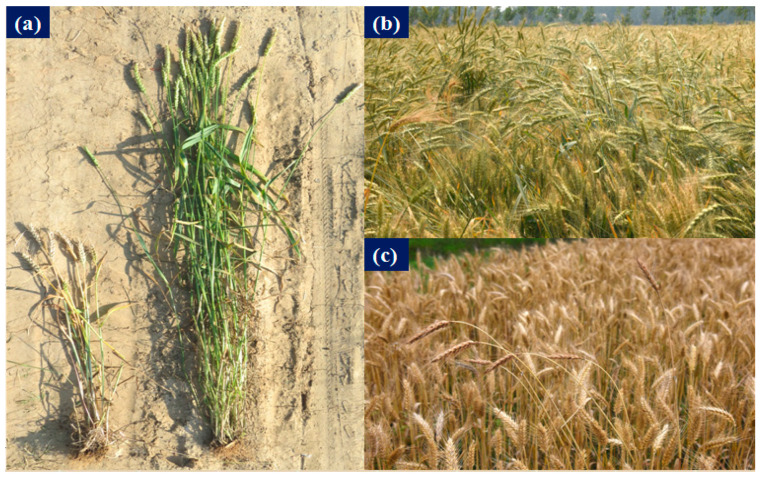
Comparative aspects of the morphology of cultivated wheat and volunteer wheat (original): (**a**) left: cultivated wheat; right: volunteer wheat; (**b**) the occurrence of volunteer wheat in wheat planting areas; (**c**) the growth situation of volunteer wheat in a field.

Volunteer wheat, also known as “wild wheat” or “semi-wheat” [1], is commonly found in the main wheat-producing areas of China, especially in the Henan, Hebei, and Shanxi Provinces (Figure 1) [2]. After maturing and germinating together with cultivated wheat sown the previous autumn, volunteer wheat has been found to be dormant in the soil during summer, which is the origin of its name. Consistent with cultivated wheat, volunteer wheat possesses 42 chromosomes [3]. The occurrence and the development of volunteer wheat have been shown to be similar to those of weedy rice (*Oryza sativa* L.) [4,5]. Previous studies have revealed differences in morphology, yield, and quality between volunteer wheat and cultivated wheat. For example, the plant height of volunteer wheat was higher than that of cultivated wheat, while the ear length and stem diameter, as well as yield and quality, were significantly smaller and lower than those of cultivated wheat [6]. Furthermore, volunteer wheat has a long dormancy period, and illumination with white light prolonged the dormancy time by reducing the degradation rate of endogenous abscisic acid (ABA) in long-dormancy volunteer wheat [7]. Consequently, the long sunshine duration allows volunteer wheat to pass through the summer in a dormant state, which leads volunteer wheat to emerge with cultivated wheat in autumn [7].

Because of these dormancy and growth characteristics, volunteer wheat hybridizes with cultivated wheat easily and competes for resources needed for survival, such as water, fertilizer, and light, which has caused serious damage to cultivated wheat production in China (Figure 1). Previous research showed that the frequency of volunteer wheat in cultivated wheat-producing areas was 50.3–92.1% [6]. In addition, the photosynthetic characteristics, chlorophyll fluorescence parameters, pigment, and malondialdehyde contents of volunteer wheat were found to be higher than those of cultivated wheat [7], which resulted from the severe reduction in yield and the deterioration of breeding [3,8]. Furthermore, volunteer wheat’s slender and tall stems easily cause lodging among wheat crops, leading to a reduction in the yield of cultivated wheat [8]. However, volunteer wheat is extremely difficult to control due to its similarity to cultivated wheat. The easiest methods for controlling volunteer wheat also harm cultivated wheat, as the chemical herbicides used in wheat fields are not selective for volunteer wheat, making chemical weeding difficult. Their use also increases the possibility that volunteer wheat could become a “superweed” in cultivated wheat fields [9]. Currently, production relies heavily on controlling volunteer wheat blends and manual pulling to prevent and reduce the occurrence of companion wheat, which requires a lot of labor and material resources. Moreover, studies have revealed that volunteer wheat is a key source of infection for some fungal diseases that affect cultivated wheat. For example, *Blumeria graminis* f.sp. *tritici* can infect volunteer wheat in summer, which then infects the cultivated wheat sown in autumn with wheat powdery mildew [10]. Therefore, it is necessary to develop effective prevention and control measures for volunteer wheat.

Generally, a high percentage of germination is considered critical for a weed’s rapid spread. Seed germination plays a key role in plant growth and is affected by many environmental factors, such as temperature, soil salinity, soil osmotic potential, pH, soil seeding depth, light, and more [5,11,12,13]. Temperature influences the germination of seeds by activating metabolic processes in which different enzymes participate, especially at varying temperatures, which was shown to be an important ecological factor for the seed germination of *Avena fatua* L. (*A. fatua*), *Bromus catharticus,* and *Aegilops tauschii coss* in one study [14]. However, light had less effect on the germination of these plants [14,15,16]. Salt and osmotic stresses inhibited the germination of some weeds and affected the distribution and the expansion of weed populations in a field in another study [17]. Previous research showed that the germination percentage of *A. fatua* decreased significantly when the salt concentration exceeded 80 mmol·L^−1^ and that *A. fatua* seeds could not germinate when the salt concentration reached 320 mmol·L^−1^ [18]. In addition, seeding depth is also a decisive factor for successful germination, as different tillage methods lead to the seeds of weeds being found in different soil layers with different germination rates.

In China, the damage caused by volunteer wheat is becoming increasingly serious for crop production. Determining the germination and the emergence characteristics of volunteer wheat is of great significance for its control. To date, there is limited information available on the effects of environmental conditions on the adaptability and germination characteristics of volunteer wheat. In the present study, we intended to comparatively study the germination of volunteer and cultivated wheat under the influence of temperature, osmotic potential, salt stress, seeding depth, and soaking. The results could provide a basis for future studies concerning the control of volunteer wheat.

## 2. Materials and Methods

### 2.1. Grain Source

Long-dormancy volunteer wheat (W171) and short-dormancy volunteer wheat (W17) grains were collected in May 2017 from cultivated-wheat-producing areas in China. W171 is a volunteer wheat with a long dormancy of 11 weeks (w) and W17 is a volunteer wheat with a short dormancy of only 1 week (w) [7]. The areas from which the volunteer wheat grains were collected are shown in Table 1. Cultivated wheat (W0, Bainong Ak58, harvested in June 2018) grains were purchased from Qiule Seeds Technology Co., Ltd. Zhengzhou, Hennan Province, China. The volunteer wheat grains were collected from ridges between rows of cultivated wheat. The collected volunteer wheat W171 and W17 grains were sown in autumn 2017 and harvested in June 2018, and then the cleaned grains were stored in paper bags at room temperature (20 ± 5 °C) until further use.

### 2.2. Germination Experiment

Germination experiments were conducted in 90 mm diameter Petri dishes (Shanghai Wuyi Glass Instrument Manufacture, Shanghai, China). Each Petri dish was lined with two pieces of Whatman No.1 filter paper (GE Healthcare Bio-Sciences, Pittsburgh, PA, USA), and 5 mL of distilled water (pH = 6.8) or the test solution was added to them. Twenty grains were placed in each Petri dish. Petri dishes sealed with Parafilm were placed in incubators (GXZ-300B, Jiangnan Instrument Manufacture, Ningbo, China) at fluctuating day/night temperatures of 25/15 °C and with 88 µmol m^−2^s^−1^ of illumination under 12 h light/12 h dark conditions. Grains were considered to have germinated when the radicle was longer than 2 mm [19]. The germinated grains were counted every 2 days until the 16th day after sowing, and the germinated grains were removed. Each treatment was repeated 4 times. The vigor of non-germinated grains was tested using 0.5% 2,3,5-triphenyltetrazolium chloride at 30 °C. After 4 h, grain embryos showing a pink to red color were considered living [20].

### 2.3. Effects of Temperature on Germination

Grains were germinated under constant temperatures (day/night temperatures: 5/5, 10/10, 15/15, 20/20, 25/25, 30/30, 35/35, and 40/40 °C) and fluctuating temperatures (day/night temperatures: 35/25, 30/20, 25/15, 20/10, and 15/5 °C) under 12 h light/12 h dark conditions. All other conditions were the same as the germination experiment.

### 2.4. Effects of Osmotic Potential on Germination

Using polyethylene glycol (PEG) 8000 to simulate water stress, the grains were placed in solutions with water potentials of 0, −0.1, −0.2, −0.4, −0.6, −0.8, −1.0, and −1.2 MPa, which was achieved by dissolving 0, 79.55, 118.94, 175.00, 218.14, 254.55, 286.65, and 315.67 g of PEG 8000 in 1 L distilled water, respectively [21]. All other conditions were the same as the germination experiment.

### 2.5. Effects of Salt Stress on Germination

Grains were germinated in Petri dishes that contained 5 mL of sodium chloride (NaCl) solutions of different concentrations (0, 50, 100, 150, 200, 250, 300, and 400 mmol·L^−1^). All other conditions were the same as the germination experiment.

### 2.6. Grain Longevity after Soaking in Water

Grains were placed in 100 mL beakers containing distilled water at 20 °C for 0, 6, 12, 24, and 36 h. Then, the grains were removed from the beakers and air-dried. The grain vigor was determined by placing them in Petri dishes for germination. All other conditions were the same as the germination experiment.

### 2.7. Effects of Seeding Depth on Seedling Emergence

A total of 30 grains were sown in plastic pots (10 cm diameter and 12 cm depth) at a depth of 0 cm (soil surface) or covered with soil (2:1 (wt/wt) sand:soil, pH 6.6, and 1.4% organic matter) to a depth of 2, 4, 6, 8, or 10 cm. Then, the pots were randomly placed in incubators. The optimal moisture levels of the pots were maintained as needed. All other conditions were the same as in the germination experiment. Then, the number of germinated grains with coleoptiles that could be distinguished visually were measured daily until 26 d after sowing. Finally, the failure of the non-emerged grains was determined based on failure to germinate or failure of the coleoptile to break through the soil surface. In addition, the grain bank in the soil was determined using non-grained pots as controls. After 30 d of sowing, no volunteer wheat was found in the control pots, indicating that there was no grain bank of volunteer wheat in the soil.

### 2.8. Statistical Analysis

Data aggregation and calculation were performed using Excel 2010 (Microsoft, Seattle, WA, USA) software. Non-linear fitting of the grain germination process and germination status under osmotic and salt stress was conducted using SigmaPlot 10.0 (Systat Software Inc., SanJose, CA, USA) software. Significance analysis of the statistics was performed using SPSS 18.0 (IBM, Chicago, IL, USA) software (LSD method at the *p* < 0.05 level). The correlations between osmotic potential, salt stress, grain longevity, seeding depth, and germination percentage were analyzed using the Pearson correlation.

The equation for calculating the percentage of germination (emergence) was as follows:

The germination percentage = [Number of germinating (emerged) grains/Total number of grains] × 100%

The non-linear fitting equation for the grain germination and emergence processes was as follows [22]:

*E* = *a*/{1 + exp[− (*x* − *t*_50_)/*b*}

where *E* is the total seedling germination or emergence rate (%) at time *x*, *a* is the maximum germination or emergence rate (%), *t*_50_ is the time taken to reach 50% of the final germination or emergence, and *b* indicates the slope of *t*_50._

The mean germination time (*MGT*) or mean emergence time (*MET*) and germination index (*GI*) or emergence index (*EI*) are shown in the following equation [23]:

*MGT* (*MET*) = ∑(*N_i_* × *D_i_*)/∑*N_i_*

*GI* (*EI*) = ∑*N_i_*/*D_i_*

where *N_i_* is the number of grains germinating or emerging at time *D_i_* days.

The equation of germination rates (%) obtained at different concentrations of NaCl and osmotic potentials is *G* = *G*_max_/[1 + (*x*/*x*_50_)^g^], where *G* represents the total percentage germination (%) at NaCl concentration or osmotic potential *x*, *G*_max_ is the maximum germination (%), *x*_50_ is the NaCl concentration or osmotic potential for 50% inhibition of the maximum germination, and ^g^ indicates the slope of the equation.

## 3. Results

### 3.1. Effects of Temperature on Germination

**Figure 2:**
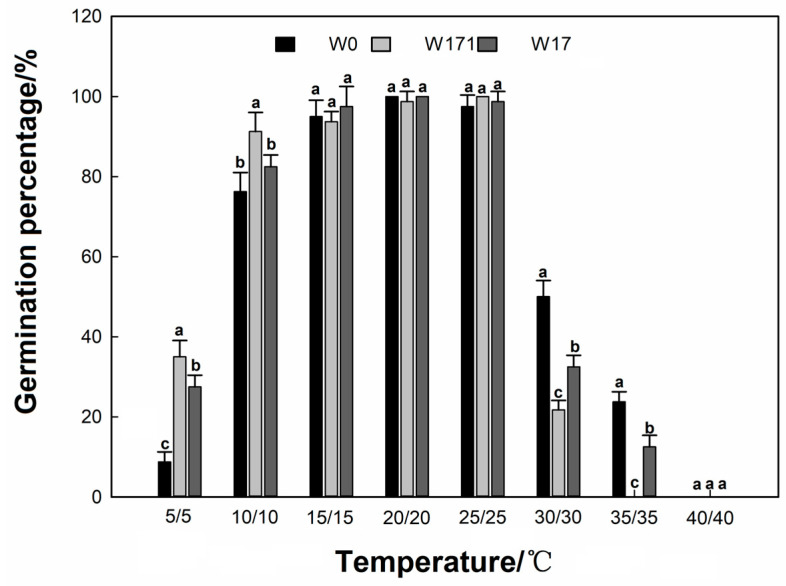
Effects of constant temperatures on the germination of cultivated wheat and volunteer wheat. Different letters indicate significant differences between the W0, W171, and W17 wheats under the same treatment at the 0.05 level.

At constant temperatures of 15 °C, 20 °C, and 25 °C, the germination percentages of the cultivated wheat as well as of the long- and short-dormancy volunteer wheat were above 90%, with these data showing no significant differences among the groups (*p* > 0.05). As the temperature decreased to a constant temperature of 5 °C, the germination percentages of the wheat decreased substantially for all three varieties, and the percentage of the decline in the volunteer wheat was significantly higher than that of the cultivated wheat (*p* < 0.05), where the value for cultivated wheat was as low as 8.75%, and those for long- and short-dormancy volunteer wheat were 35% and 27.5%, respectively (Figure 2), whereas the germination time (*t*_50_ and MGT) of both long- and short-dormancy volunteer wheat were significantly lower than that of cultivated wheat (*p* < 0.05) (Table 2). As the temperature increased to a constant temperature of 30 °C, the germination percentages of cultivated wheat decreased substantially across all three varieties, with the germination percentages of long- and short-dormancy volunteer wheat being significantly lower than that of cultivated wheat (50%, *p* < 0.05), while the germination times (*t*_50_ and MGT) of long-dormancy volunteer wheat were significantly higher than that of cultivated wheat (*p* < 0.05) (Table 2). At a constant temperature of 35 °C, the germination percentage of the cultivated wheat decreased to 25%, while the long-dormancy volunteer wheat did not germinate. At a constant temperature of 40 °C, all three varieties of wheat failed to germinate. Compared to cultivated wheat, the results under constant temperature regimes of *t*_50_, MGT, and GI (Table 2) showed that high temperature above 30 °C significantly inhibited the germination of volunteer wheat compared to cultivated wheat, but low temperature below 10 °C had little effect on the germination activity of long- and short-dormancy volunteer wheats.

**Figure 3:**
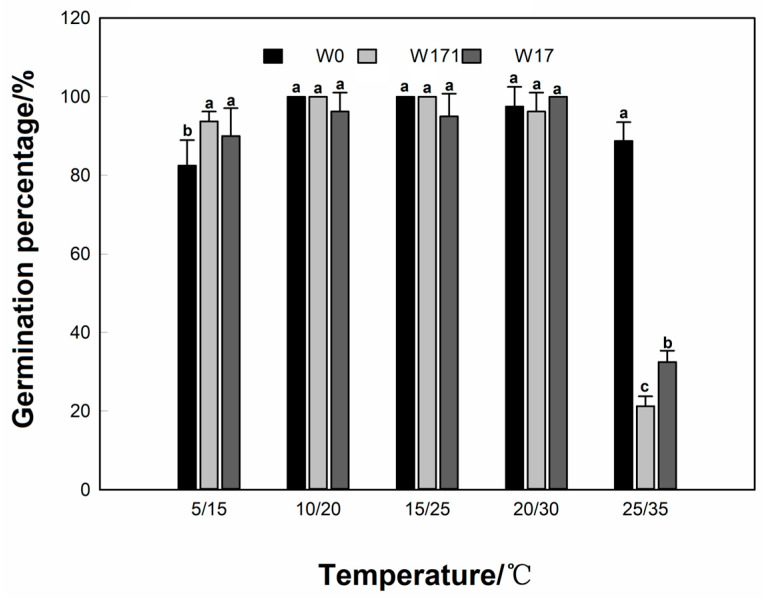
Effects of fluctuating temperatures on the germination of cultivated wheat and volunteer wheat. Different letters indicate significant differences between the W0, W171, and W17 wheats under the same treatment at the 0.05 level.

At variable temperatures of 10 °C/20 °C, 15 °C/25 °C, and 20 °C/30 °C, the germination percentages of the cultivated wheat and the long- and short-dormancy volunteer wheat were above 95%, and the data showed no significant changes among the three groups with the same treatment (*p* > 0.05). As the temperature decreased to 5 °C/15 °C, the germination percentage of the cultivated wheat decreased to 82.5%, and that of the short-dormancy volunteer wheat decreased to 90% (Figure 3), while the germination percentage of the long-dormancy volunteer wheat did not decrease significantly. Meanwhile, the germination times (*t*_50_ and MGT) of the long- and short-dormancy volunteer wheats were significantly lower than that of the cultivated wheat, whereas the GI values were significantly higher than that of the cultivated wheat (*p* < 0.05), indicating that the long- and short-dormancy wheats had a stronger germination ability than the cultivated wheat at low temperatures. As the temperature increased to 25 °C/35 °C, the germination percentage of the cultivated wheat did not decrease significantly. However, the germination percentages of the long- and short-dormancy volunteer wheats decreased to 21.3% and 32.5%, respectively, and the GI values were significantly lower than that of the cultivated wheat (*p* < 0.05), indicating that the germination ability of the volunteer wheats at high temperatures was weaker than that of the cultivated wheat (Table 2).

### 3.2. Effects of Osmotic Potential on Germination

**Figure 4:**
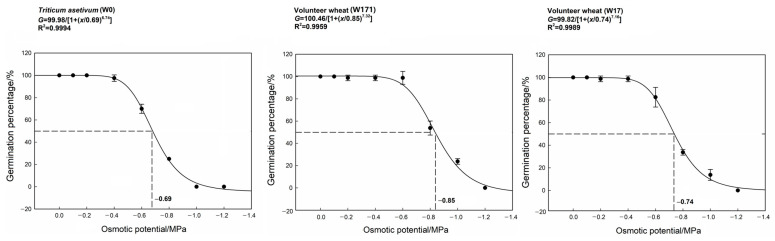
Effects of osmotic potential on the germination of cultivated wheat and volunteer wheat. Error bars represent standard errors of the means. Values represent the mean of four replications with 20 seeds per replication.

With decreasing osmotic potential, the germination percentages of both the cultivated and volunteer wheats showed decreasing trends. When the osmotic potential was reduced to −0.6 MPa, the germination percentages of the cultivated and short-dormancy volunteer wheats decreased significantly, while only the long-dormancy volunteer wheat decreased significantly at −0.8 MPa. The germination of the cultivated wheat was completely inhibited at −1.0 MPa. The osmotic potential was significantly correlative with the germination percentage of each variety (W0: r = 0.952, *p* < 0.001; W171: r = 0.928, *p* < 0.001; W17: r = 0.942, *p* < 0.001). The osmotic potentials for 50% inhibition of the maximum germination were −0.69, −0.85, and −0.74 MPa for cultivated, long-dormancy volunteer, and short-dormancy volunteer wheats, respectively (Figure 4), which indicated the tolerance of volunteer wheat to reduced osmotic potential was greater than that of cultivated wheat, with long-dormancy volunteer wheat able to withstand lower osmotic potential during germination. The decrease in osmotic potential was accompanied by an increasing trend in the germination time (*t*_50_ and MGT) and a decreasing trend in the GI for all three varieties of cultivated wheat (Table 3). At lower osmotic potentials (−0.2, −0.4, −0.6, −0.8 MPa), the *t*_50_ of the long-dormancy volunteer wheat was significantly lower than that of the cultivated wheat, and the GI was significantly higher than that of the cultivated wheat and short-dormancy volunteer wheat. The *t*_50_ values of the short-dormancy volunteer wheats were lower than those of the cultivated wheat at −0.6 and −0.8 MPa osmotic potentials, while the GI was conversely higher. These results also indicated that the volunteer wheats were more tolerant of lower osmotic potentials during germination, especially the long-dormancy volunteer wheat, which was most tolerant of lower osmotic potentials (Table 3). 

### 3.3. Effects of Salt Stress on Germination

**Figure 5:**
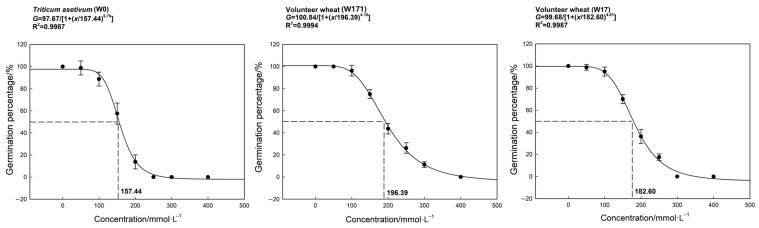
Effects of NaCl concentration on the germination of cultivated and volunteer wheat. Error bars represent standard errors of the means. Values represent the mean of four replications with 20 seeds per replication.

The germination percentages of both the cultivated wheat and the volunteer wheat showed decreasing trends with increasing concentrations of NaCl, and no grains could germinate normally at NaCl concentrations of 250, 400, and 300 mmol·L^−1^ for cultivated, long-dormancy volunteer, and short-dormancy volunteer wheats, respectively. The concentrations of NaCl for 50% inhibition of the maximum germination were 157.44, 196.39, and 182.60 mmol·L^−1^ for cultivated, long-dormancy volunteer, and short-dormancy volunteer wheats, respectively (Figure 5), which indicated that long-dormancy volunteer wheat had the strongest tolerance to salt stress. After combining the germination percentages from the cultivated and volunteer wheats at different concentrations of NaCl, it could be concluded that the tolerance of the volunteer wheats to salt stress during germination was significantly stronger than that of cultivated wheat, and the long-dormancy volunteer wheat was the most tolerant to salt stress. The salt stress was significantly correlative with the germination percentage of each variety (W0: r= −0.916, *p* < 0.001; W171: r= −0.965, *p* < 0.001; W17: r = −0.951, *p* < 0.001). The germination time (*t*_50_ and mgt) of the three cultivated wheat varieties tended to increase and the GI tended to decrease with increasing salt concentration (Table 4). At NaCl concentrations of 50–200 mmol·L^−1^, the *t*_50_ values of both long- and short-dormancy volunteer wheats were significantly lower than that of the cultivated wheat. The GI values of both the long- and short-dormancy volunteer wheats were significantly higher than that of the cultivated wheat, indicating that the tolerance of the volunteer wheats to salt stress during germination was significantly stronger, with long-dormancy volunteer wheat having the strongest tolerance to salt stress.

### 3.4. Grain Longevity after Soaking in Water

**Figure 6:**
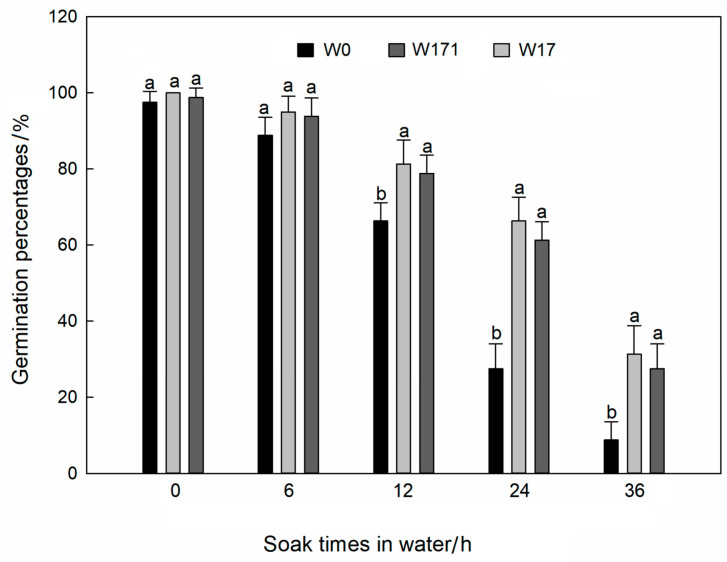
The germination of volunteer wheat and cultivated wheat grains after soaking in water for different lengths of time. Error bars represent standard errors of the means. Different letters indicate significant differences between the W0, W171, and W17 wheats under the same treatment at 0.05 level.

The germination percentages of both the cultivated wheat and the volunteer wheat showed a gradual decrease with increasing soaking times in water (Figure 6). When the soaking time increased to 12–36 h, the germination percentages of the volunteer wheats were significantly higher than that of the cultivated wheat (*p* < 0.05). Specifically, the germination percentages of long-dormancy volunteer wheat could reach 1.23–3.57 times that of cultivated wheat, while those of short-dormancy volunteer wheat could reach 1.19–3.14 times that of cultivated wheat. This result indicated the volunteer wheat had stronger secondary germination ability than the cultivated variety. The soaking time was significantly correlative with the secondary germination of each variety (W0: r = −0.982, *p* < 0.001; W171: r = −0.965, *p* < 0.001; W17: r = −0.976, *p* < 0.001).

### 3.5. Effects of Seeding Depth on Seedling Emergence

**Figure 7:**
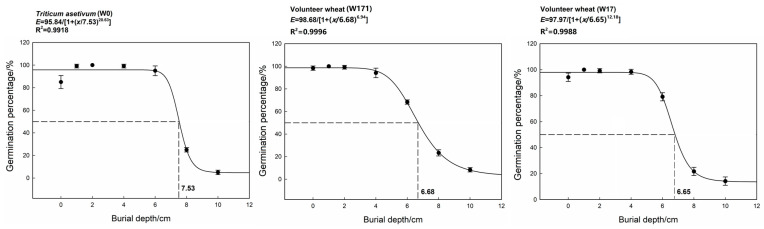
Effects of seeding depth on the seedling emergence of cultivated wheat and volunteer wheat. Error bars represent standard errors of the means. Values represent the mean of four replications with 30 seeds per replication.

With increased seeding depth, the emergence percentages of the cultivated and volunteer wheats decreased, and the long- and short-dormancy volunteer wheats could not emerge normally at seeding depths up to 10 cm. The seeding depth was significantly correlative with the germination percentage of each variety(W0: r = −0.816, *p* < 0.001; W171: r = −0.936, *p* < 0.001; W17: r = −0.895, *p* < 0.001). The seeding depths for 50% inhibition of the maximum emergence were 7.53 cm, 6.68 cm, and 6.65 cm for the cultivated, long-dormancy volunteer, and short-dormancy volunteer wheats, respectively, indicating that the emergence ability of the volunteer wheat was significantly weaker than that of the cultivated wheat (Figure 7). When the seeding depth was 4–8 cm, the *t*_50_ values and MET of the long- and short-dormancy volunteer wheats were significantly higher than those of the cultivated wheat, and the EIs of the long- and short-dormancy volunteer wheats were significantly lower than that of the cultivated wheat, indicating that the emergence ability of the volunteer wheat was weaker than that of the cultivated wheats (Table 5).

## 4. Discussion

In recent years, volunteer wheat has spread rapidly in the main wheat-producing regions near the Yellow and Huaihai Seas of China, significantly affecting the normal growth and production of cultivated wheat [24,25]. Volunteer wheat shows strong competitiveness in wheat-producing areas and significantly impacts the production of the cultivated wheat by overwhelming the space. Previous research has shown that the germination and emergence of weeds in fields are closely related to the external environmental conditions, such as temperature, seeding depth, and more [25,26]. Studies have shown that malignant weeds of wheat fields, such as *Alopecurus myosuroides* Huds., *Alopecurus aequalis* Sobol., and *Aegilops tauschii* Coss., all have high germination and emergence adaptability [24,27]. Currently, it has been reported that white light has no effect on the germination of volunteer wheat grains after dormancy release; however, it can prolong the dormancy time of volunteer wheat in dormancy [9]. 

The present study showed that volunteer wheat has high germination adaptability at constant temperatures from 15 °C to 25 °C, variable temperatures from 5 °C/15 °C to 20 °C/30 °C, osmotic potential from −0.2 to −0.6 MPa, and salinity from 50 to 200 mmol**·**L^−1^, which is similar to the germination adaptability seen in goatgrass at 10–30 °C [24]. The germination percentage of volunteer wheat was significantly correlative with osmotic potential, salt stress, grain longevity, and seeding depth. Compared to cultivated wheat, the volunteer wheats had higher germination percentages, shorter MGTs, and better GIs under low temperature conditions of 5 °C, among which the tolerance of the long-dormancy volunteer wheat to low temperatures was stronger than that of the short-dormancy volunteer wheat. This result indicates that the stronger tolerance of volunteer wheat to low temperatures could be one of its advantages in higher latitudes. A similar phenomenon was also found in weedy rice, which adapted to the temperature range for rice germination and had a wider range of tolerable temperatures than rice [5,28]. Conversely, the germination ability of the volunteer wheat was weaker than that of cultivated wheat at high temperatures above 30 °C, which might result in the fact that volunteer wheat does not germinate during the summer at high temperatures. The secondary germination ability of volunteer wheat was significantly higher than that of cultivated wheat after a soaking time greater than 12 h. Qin et al. [29] reported that the germination percentages of graminaceous grass *Cynodon dactylon* (L.) Pers. decreased as the time that the seeds were soaked in water increased. The germination ability of the seeds of different species has been shown to vary after soaking in water [30]. Under osmotic and salt stress, the germination ability of the long-dormancy volunteer wheat was the strongest, while that of the cultivated wheat was the weakest. The germination percentages of the long-dormancy volunteer wheat at the NaCl concentration of 300 mmol·L^−1^ were still above 10%, which enables the volunteer wheat grains to be fully adaptable to most field characteristics. Most weeds have been proven to be highly salt- and drought-tolerant [23,31,32]. Wang et al. [16] reported that more than 80% of *Aegilops tauschii* seeds germinated at 40 mol·L^−1^ salinity, but between 10% and 30% of the seeds could even germinate at 400 mol·L^−1^ salinity.

Seeding depth is one of the important factors affecting the emergence of weeds, such as *Alopecurus myosuroides* Huds., *Alopecurus aequalis* Sobol., *Aegilops tauschii* Coss., *Cucumis melo* L. var. *agrestis* Naud., *Polypogon fugax* Nees ex Steud., and *Oryza sativa* L. [23,26,31,32]. The suitable germination depth of *Bromus japonicas* was 0.5–3.5 cm, that of *Aegilops tauschii* was 0.5–6.0 cm, and that of *Avena Fatua* was the widest, ranging from 0 to 20.0 cm [33]. This study showed that the long- and short-dormancy volunteer wheat varieties could not emerge normally when the seeding depth was greater than 8 cm, and the emergence ability was weaker than that of cultivated wheat, which might be related to the smaller thousand-grain-weight of volunteer wheat grains [1] compared to cultivated wheat and, therefore, the smaller nutrient stores. The size of seeds also determines the seed germination depth by determining their soil-pushing ability: the smaller the seed, the less energy it has stored, and the more difficult it is to germinate [34]. Moreover, deep tilling of the land has been a proven and effective measure for controlling the spread of volunteer wheat, and this result was consistent with results for other weeds [35,36,37,38], with a significant reduction in germination with increasing soil depth [27,31,39].

Furthermore, volunteer wheat growing in the primary wheat-producing regions adjacent to the Yellow and Huaihai Seas in China exhibits robust dormancy, photosynthetic capacity, and light energy conversion efficiency [7,9]. The no-till farming model, commonly employed in corn–wheat rotation systems in recent years, leads to the presence of volunteer wheat grains on the soil surface that cannot be regulated by seeding depth. The characteristics of volunteer wheat, such as its high-temperature germination inhibition, strong secondary germination, and dormancy traits, and the effect of white light [9] ensure that its grains remain dormant in the field during hot and humid summers after the wheat harvest. Consequently, these grains only germinate in the suitable temperatures of autumn, which may further facilitate the spread of volunteer wheat. The strong germination and emergence of volunteer wheat grains, coupled with their dormancy characteristics, necessitate timely and effective prevention and control measures to curb the spread of this malignant weed in winter wheat planting areas.

## 5. Conclusions

The adaptability of volunteer wheat was superior to that of cultivated wheat in terms of low temperature tolerance and osmotic and salt stress resistance. Additionally, volunteer wheat exhibited a higher secondary germination ability following water immersion. However, its adaptability weakened at high temperatures exceeding 30 °C. The adaptability of volunteer wheat grains to seeding depth was inferior to that of cultivated wheat. Thus, deep sowing may prove to be a viable strategy in the prevention and management of volunteer wheat. The findings of this study indicate that the grains of volunteer wheat exhibit superior adaptability and germination capabilities compared to those of cultivated wheat, thereby elucidating the significant impact caused by volunteer wheat on cultivated wheat production. The findings of this study can serve as a foundation for managing volunteer wheat.

## Figures and Tables

**Table 1:** General data on cultivated wheat and volunteer wheat [7].

Phenotypic Forms of Wheat	Regions/Variety	Awn Type	Mean Plant Height (cm)	Mean Number of Grains Per Spike	1000-Grain-Weight (g)	Number of Weeks for Dormancy Period (w)
Volunteer wheat—W171	Henan, Minquan (N 34°39′, E 114°48′)	No awn	121	42	31.4	11
Volunteer wheat—W17	Henan, Zhengyang (N 33°01′, E 114°02′)	Long awn	126	41	31.6	1
Cultivated wheat—W0	Henan (Bainong Ak58)	Long awn	70	32	44.6	0

**Table 2:** Effects of temperature on the t_50_, MGT, and GI of cultivated wheat and volunteer wheat.

Treatment		t_50_/d			MGT/d			GI		
		W0	W171	W17	W0	W171	W17	W0	W171	W17
Constant temperature regimes/(°C/°C)	5/5	11.57 ± 0.81 a	8.99 ± 0.63 b	9.37 ± 1.25 b	15.77 ± 0.45 a	14.75 ± 0.08 ab	14.28 ± 0.43 b	0.60 ± 0.20 b	3.11 ± 0.27 a	2.78 ± 0.23 a
	10/10	7.61 ± 0.49 a	5.75 ± 0.15 b	5.51 ± 0.27 b	13.89 ± 0.15 a	13.17 ± 0.12 b	13.21 ± 0.08 b	8.65 ± 0.18 c	12.80 ± 0.89 a	11.30 ± 0.27 b
	15/15	5.88 ± 0.74 a	4.18 ± 0.16 b	5.66 ± 0.11 a	13.16 ± 0.23 a	12.53 ± 0.04 b	13.03 ± 0.07a	13.50 ± 0.73 c	16.32 ± 0.27 a	14.56 ± 0.44 b
	20/20	2.00 ± 0.02 a	1.90 ± 0.11 a	1.80 ± 0.20 a	11.49 ± 0.08 a	11.34 ± 0.09 b	11.38 ± 0.02 ab	24.29 ± 0.68 b	25.36 ± 0.44 a	25.35 ± 0.13 a
	25/25	2.07 ± 0.17 b	2.21 ± 0.14 b	2.57 ± 0.06 a	11.73 ± 0.06 b	11.72 ± 0.07 b	11.91 ± 0.04 a	22.15 ± 0.53 a	22.56 ± 0.56 a	20.99 ± 0.49 b
	30/30	7.47 ± 0.68 b	10.26 ± 0.95 a	10.01 ± 0.72 a	14.16 ± 0.26 b	15.07 ± 0.49 a	13.90 ± 0.20 b	5.18 ± 0.16 a	1.76 ± 0.19 c	3.58 ± 0.30 b
	35/35	10.00 ± 0.73 a	-	11.23 ± 0.95 a	15.24 ± 0.23 a	-	15.09 ± 0.54 a	1.82 ± 0.08 a	-	0.98 ± 0.17 b
	40/40	-	-	-	-	-	-	-	-	-
Fluctuating temperature regimes/(°C/°C)	5 °C/15	7.49 ± 0.61 a	6.41 ± 0.44 b	5.37 ± 0.36 c	14.12 ± 0.18 a	13.52 ± 0.16 b	13.18 ± 0.13 c	8.63 ± 0.34 c	11.81 ± 0.63 b	12.43 ± 0.46 a
	10 °C/20	2.26 ± 0.23 a	2.48 ± 0.24 a	2.20 ± 0.16 a	11.70 ± 0.12 a	11.83 ± 0.09 a	11.73 ± 0.08 a	22.64 ± 0.92 a	21.71 ± 0.74 a	21.88 ± 0.94 a
	15 °C/25	1.60 ± 0.26 a	1.71 ± 0.21 a	1.87 ± 0.06 a	11.38 ± 0.07 b	11.46 ± 0.05 ab	11.50 ± 0.02 a	25.54 ± 0.54 a	24.73 ± 0.55 a	23.08 ± 1.01 b
	20 °C/30	5.94 ± 0.35 c	7.71 ± 0.18 a	6.66 ± 0.43 b	13.19 ± 0.13 c	13.90 ± 0.06 a	13.48 ± 0.09 b	13.75 ± 0.27 a	10.85 ± 0.14 c	12.86 ± 0.30 b
	25 °C/35	9.89 ± 0.34 ab	8.87 ± 1.39 b	11.38 ± 1.18 a	15.06 ± 0.05 a	14.45 ± 0.18 b	15.08 ± 0.13 a	7.13 ± 0.21 a	1.97 ± 0.32 c	2.56 ± 0.18 b

Note: Values represent mean ± SE. Different letters indicate significant differences between the W0, W172, and W17 wheats under the same treatment at the 0.05 level.

**Table 3:** Effects of osmotic potential on t_50_, MGT, and GI of cultivated wheat and volunteer wheat.

Osmotic Potential/MPa	t_50_/d			MGT/d			GI		
	W0	W171	W17	W0	W171	W17	W0	W171	W17
0	1.64 ± 0.33 a	1.75 ± 0.13 a	1.85 ± 0.16 a	11.61 ± 0.08 a	11.50 ± 0.06 a	11.55 ± 0.10 a	25.29 ± 0.84 a	24.73 ± 0.43 ab	24.16 ± 0.66 b
−0.1	2.05 ± 0.12 a	1.89 ± 0.09 a	1.91 ± 0.15 a	11.96 ± 0.19 a	11.78 ± 0.08 b	11.88 ± 0.08a b	23.41 ± 0.60 a	24.29 ± 0.54 a	23.98 ± 0.75 a
−0.2	2.77 ± 0.57 a	2.31 ± 0.20 b	2.57 ± 0.21 ab	12.74 ± 0.08 a	12.41 ± 0.16 b	12.71 ± 0.23 a	21.08 ± 1.36 b	21.99 ± 1.17 a	21.19 ± 1.16 ab
−0.4	4.63 ± 0.27 a	3.91 ± 0.37 b	4.81 ± 0.49 a	15.72 ± 0.56 b	17.91 ± 1.41 a	16.39 ± 1.61 b	15.72 ± 0.56 b	17.91 ± 1.41 a	16.39 ± 1.61 b
−0.6	7.94 ± 0.22 a	5.83 ± 0.10 c	6.35 ± 0.34 b	14.21 ± 0.20 a	13.39 ± 0.07 c	13.74 ± 0.13 b	7.29 ± 0.83 c	11.67 ± 0.71 a	9.67 ± 0.95 b
−0.8	9.52 ± 1.54 a	7.89 ± 0.18 b	7.84 ± 1.41 b	15.04 ± 0.60 a	14.15 ± 0.10 b	14.35 ± 0.44 b	2.07 ± 0.36 c	4.16 ± 0.55 a	2.62 ± 0.50 b
−1.0	-	13.76 ± 0.52 a	13.99 ± 0.06 a	-	17.21 ± 0.40 a	17.56 ± 0.34 a	-	1.02 ± 0.15 a	0.55 ± 0.24 b
−1.2	-	-	-	-	-	-	-	-	-

Note: Values represent mean ± SE. Different letters indicate significant differences between the W0, W172, and W17 wheats under the same treatment at the 0.05 level.

**Table 4:** Effects of NaCl concentration on t_50_, MGT, and GI of cultivated and volunteer wheat.

NaCl Concentratio/ (mmol·L^−1^)	t_50_			MGT			GI		
	W0	W171	W17	W0	W171	W17	W0	W171	W17
0	1.73 ± 0.15 a	1.84 ± 0.05 a	1.86 ± 0.15 a	11.37 ± 0.11 b	11.48 ± 0.07 ab	11.53 ± 0.08 a	25.54 ± 0.94 a	24.54 ± 0.58 ab	24.16 ± 0.66 b
50	3.19 ± 0.16 a	2.25 ± 0.17 b	1.97 ± 0.19 c	12.12 ± 0.04 a	11.72 ± 0.09 b	11.66 ± 0.09 b	19.34 ± 0.33 b	22.56 ± 0.68 a	22.99 ± 0.70 a
100	5.89 ± 0.20 a	3.91 ± 0.58 b	3.52 ± 0.32 b	13.38 ± 0.05 a	12.48 ± 0.23 b	12.23 ± 0.16 c	11.58 ± 0.93 b	16.88 ± 1.01 a	18.28 ± 0.21 a
150	6.96 ± 0.28 a	5.90 ± 0.12 b	5.92 ± 0.30 b	13.82 ± 0.40 a	13.37 ± 0.21 a	13.34 ± 0.10 a	6.64 ± 0.49 b	9.79 ± 0.42 a	9.26 ± 0.64 a
200	9.25 ± 1.00 a	7.85 ± 0.48 b	8.21 ± 0.57 b	14.96 ± 0.22 a	14.34 ± 0.17 b	14.64 ± 0.18 a	1.15 ± 0.26 c	4.37 ± 0.53 a	2.96 ± 0.75 b
250	-	8.38 ± 0.41 b	12.27 ± 1.03 a	-	14.70 ± 0.16 b	16.60 ± 0.34 a	-	2.40 ± 0.52 a	0.93 ± 0.14 b
300	-	10.17 ± 0.03	-	-	11.62 ± 7.65	-	-	0.78 ± 0.19	-
400	-	-	-	-	-	-	-	-	-

Note: Values represent mean ± SE. Different letters indicate significant differences between the W0, W172, and W17 wheats under the same treatment at the 0.05 level.

**Table 5:** Effects of seeding depth on t_50_, MET, and EI of cultivated and volunteer wheat.

Seeding Depth/cm	t_50_			MET			EI		
	W0	W171	W17	W0	W171	W17	W0	W171	W17
0	2.86 ± 0.61 a	2.93 ± 0.44 a	2.87 ± 0.18 a	11.99 ± 0.16 a	12.04 ± 0.15 a	12.00 ± 0.05 a	27.03 ± 1.03 b	30.36 ± 0.73 a	29.90 ± 0.48 a
1	3.95 ± 0.21 a	3.94 ± 0.14 a	3.82 ± 0.17 a	12.50 ± 0.02 a	12.43 ± 0.06 a	12.35 ± 0.03 b	25.93 ± 0.39 c	27.00 ± 0.55 b	28.00 ± 0.15 a
2	5.71 ± 0.23 a	5.50 ± 0.27 ab	5.21 ± 0.06 b	13.31 ± 0.09 a	13.23 ± 0.07 ab	13.16 ± 0.01 b	19.84 ± 0.61 b	20.07 ± 0.09 ab	20.53 ± 0.41 a
4	6.44 ± 0.03 b	7.41 ± 0.19 a	7.90 ± 0.44 a	13.81 ± 0.06 b	14.17 ± 0.06 a	14.22 ± 0.10 a	17.04 ± 0.40 a	14.21 ± 0.67 b	14.44 ± 0.50 b
6	7.05 ± 0.14 b	9.08 ± 0.31 a	9.57 ± 0.89 a	14.00 ± 0.05 b	14.17 ± 0.06 a	14.22 ± 0.10 a	15.56 ± 0.90 a	14.21 ± 0.67 b	14.44 ± 0.50 b
8	8.62 ± 0.59 b	13.86 ± 0.42 a	13.17 ± 0.87 a	14.80 ± 0.18 b	17.07 ± 0.20 a	16.90 ± 0.31 a	3.27 ± 0.16 a	1.56 ± 0.12 b	1.54 ± 0.09 b
10	20.32 ± 1.42 a	-	-	18.88 ± 0.74 a	-	-	0.18 ± 0.08 c	-	-

Note: Values represent mean ± SE. Different letters indicate significant differences between the W0, W172, and W17 wheats under the same treatment at the 0.05 level.

## Data Availability

Not applicable.
